# The development of the GIN‐McMaster checklist extension for guideline adaptation protocol

**DOI:** 10.1002/gin2.70005

**Published:** 2024-12-06

**Authors:** Yang Song, Yuan Zhang, Yasser Amer, Andrea J. Darzi, Elie A. Akl, Pablo Alonso‐Coello, Holger J. Schünemann

**Affiliations:** ^1^ School of Medicine The Chinese University of Hong Kong Shenzhen China; ^2^ Iberoamerican Cochrane Centre (CCIb)—Biomedical Research Institute Sant Pau (IIB Sant Pau) Barcelona Spain; ^3^ Adaptation Working Group, Guidelines International Network Glasgow UK; ^4^ Department of Health Research Methods, Evidence, and Impact (HEI) McMaster University Hamilton Canada; ^5^ Pediatrics Department, Clinical Practice Guidelines and Quality Research Unit, Quality Management Department King Saud University Medical City Riyadh Saudi Arabia; ^6^ Alexandria Center for Evidence‐Based Clinical Practice Guidelines Alexandria University Alexandria Egypt; ^7^ Department of Internal Medicine American University of Beirut Beirut Lebanon; ^8^ Centro de Investigación Biomédica en Red de Epidemiología y Salud Pública (CIBERESP) Madrid Spain; ^9^ Clinical Epidemiology and Research Center (CERC), IRCCS Humanitas Research Hospital and Humanitas University Pieve Emanuele Milan Italy

**Keywords:** checklist, GIN‐McMaster checklist, GRADE, guideline adaptation, guidelines, healthcare decision‐making, local context

## Abstract

**Background:**

To ensure rigour and transparency in guideline adaptation and contextualization processes, standardized tools and methodological principles are needed. However, methodological challenges have been continuously documented in guideline adaptation processes.

**Objective:**

To develop a guideline international network (GIN)‐McMaster guidelines development checklist (GDC) extension for guideline adaptation.

**Methods:**

This project follows multiphase iterative approach, including (1) compiling a list of key methodological steps for guideline adaptation, based on scoping reviews of the current knowledge of guideline adaptation; (2) proposing methodological principles for guideline adaptation based on key methodological steps, in parallel to developing the GIN‐McMaster Guideline Development Checklist extension for adaptation (GDC‐adaptation extension) concerning the original checklist and methodological steps for adaptation; (3) iteratively refining the methodological steps through GIN Adaptation working group discussions, and GDC‐adaptation extension items through several rounds of Delphi consensus survey and (4) Public consultation and finalization. For methodological principles, this involves public consultation; for GDC‐adaptation extension, we will conduct user testing through semi‐structured interviews. Finally, we will submit the final outputs to the GIN board and seek final approval.

**Discussion:**

The identification of the key methodological principles, together with the GIN‐McMaster GDC extension for guideline adaptation and contextualization, will provide clarity in the planning and execution of adaptation, adoption and/or development of recommendations. The aim of the checklist is to improve efficiency and reduce research waste in guideline development while maintaining rigour and transparency.

## Key points

*What is already known on this topic?*

- Guideline development process should coordinate with existing source guidelines, their recommendations and evidence to avoid effort duplication and resource waste.
- Guideline adaptation can be an efficient method for developing guidelines. It incorporates source materials with multiple considerations from target settings.
- More than 15 adaptation frameworks have been developed, yet the process still faces challenges and limitations.
- Few standards and manuals address guideline adaptation methods and processes.

*What this study adds?*

- We aim to develop the guideline international network‐McMaster guidelines development checklist (GDC) in parallel with proposing the methodological principles for guideline adaptation.
- The development process of guidance and principles incorporates each other with a multistep and interactive development process.
- The GDC‐adaptation extension and fundamental methodological principles will clarify the planning and execution of adaptation, adoption and development of recommendations.

## 1 BACKGROUND

The methodology for developing clinical practice guidelines (CPG) has evolved rapidly for several decades and gradually has become more rigorous and complex. How to efficiently develop high‐quality CPGs remains challenging, though. Improving the efficiency of the guideline development process has become even more crucial nowadays, especially in scenarios when evidence is rapidly updated, like during the recent COVID‐19 pandemic.^1^, ^2^ Along with the increased trend of using key evidence and sharing similar decision‐making considerations, the guideline development process should coordinate with existing source guidelines, their recommendations and evidence before starting the de novo process. However, the decision‐making process often differs across settings to respond to different system needs. Isolated considerations on the same topic may cause confusion and conflicted healthcare recommendations and introduce resource waste.^3^, ^4^

Guideline adaptation can be an efficient alternative method for developing CPGs, considering recommendations developed in one setting to another, although it requires validation in terms of saving time and resources.^5^ In 1999, Feder et al. initially proposed guideline adaptation as a first step, to making good use of CPG.^6^ After several years,^7^ the adaptation of CPG now involves adapting (modifying), adopting (not modifying) existing recommendations or developing de novo recommendations, according to the needs and priority of the local context.^8^ However, published adapted guidelines are generally of low quality, poorly reported and not based on adaptation frameworks.^9^ The methods used for guideline adaptation vary widely across settings.^10^ Besides, organizations are facing many challenges, including but not limited to the complexity of the process, time and resource consumption, lack of guidance and being out of date.^11^ Guideline development organizations, including the World Health Organization and professional associations (e.g., American College of Physicians), have published their standards and manuals of the guideline development process to ensure rigour and transparency.^12^, ^13^ However, few of these standards and manuals address guideline adaptation methods and processes.^14^

The guideline international network (GIN)‐McMaster guidelines development checklist (GDC) supports developing and implementing trustworthy guidelines.^16^ The GIN‐McMaster GDC is designed to serve as a resource and tool for those interested in beginning, enhancing or evaluating their guideline development process. The purpose of the checklist is not to evaluate the quality assessment tools, like AGREE II^14^ and other tools that serve to assess credibility^17^ or reporting.^18^, ^19^ Rather, following the steps outlined in the checklist will ensure that key items are covered and increase the likelihood of the guideline improving credibility and reporting.^19^ The GIN‐McMaster GDC includes 146 elements across 18 topics, addressing all stages of the guideline enterprise, from planning to implementation and evaluation.^15^ It comes with existing or planned extensions, including rapid guideline development, quality assurance and improvement, equity considerations and stakeholder involvement.^2^, ^20^, ^21^ Besides, a preliminary matching of the ADOLOPMENT process to the checklist items was published in 2017.^22^

More methodological research on the optimization and standardization of the guideline adaptation methods and processes is warranted. Therefore, the GIN, along with McMaster GRADE Centre, the Barcelona GRADE Centre and other organizations, aims to put effort into facilitating the adaptation process by dealing with methodological challenges in the guideline adaptation process. Through a multiphase development method, we will identify relevant components, describe principles for guideline adaptation and develop a GIN‐McMaster GDC extension for guideline adaptation. The methodological principles, together with the adaptation extension for the GIN‐McMaster GDC, will provide clarity in the planning and execution of adaptation, adoption or contextualized de novo development of recommendations.

## 2 OBJECTIVES

To identify methodological components and propose principles for guideline adaptation and to develop the GIN‐McMaster GDC extension for guideline adaptation (GDC‐adaptation extension).

## 3 METHODS

**Figure 1:**
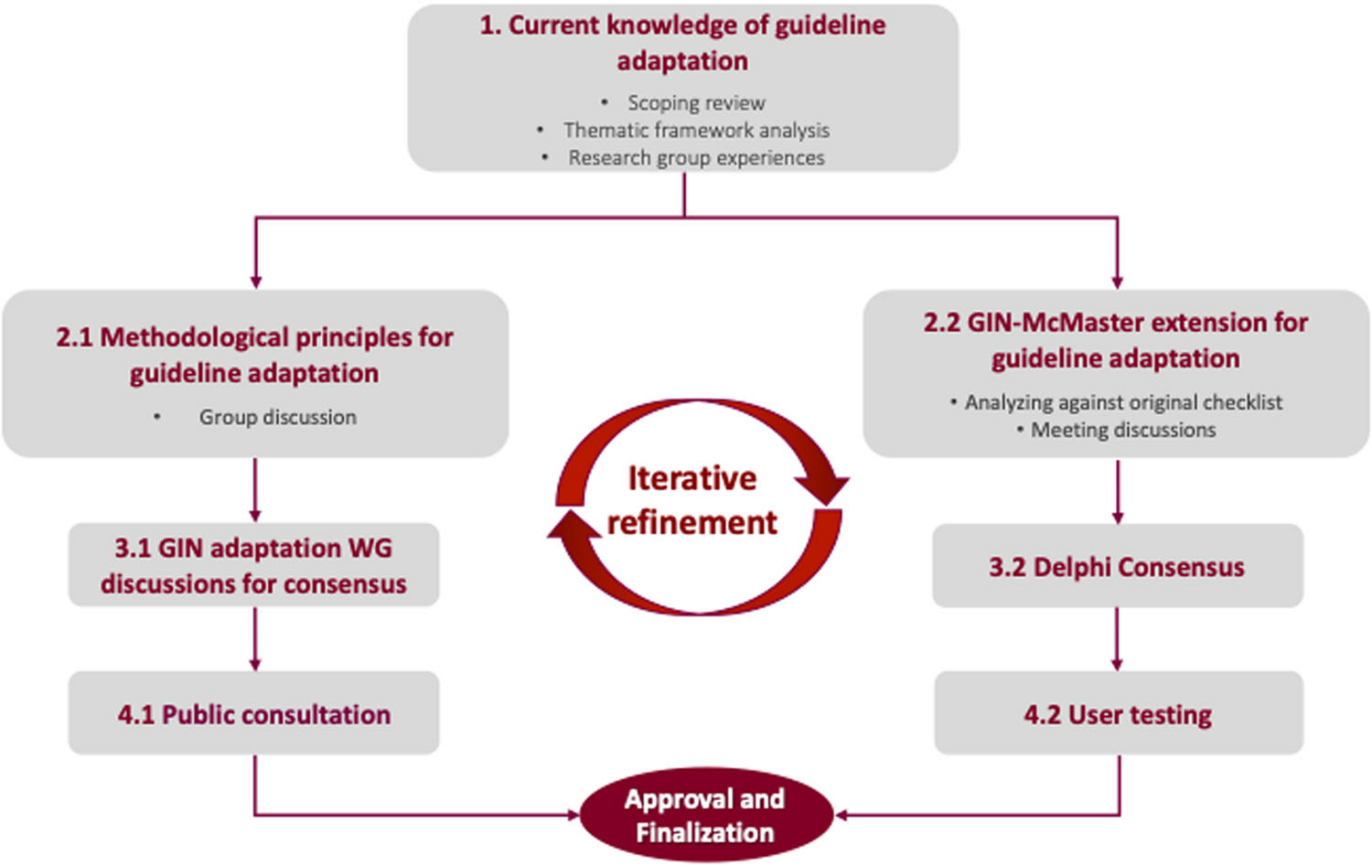
Development processes.

We will propose and optimize the GDC‐adaptation extension through a multiphase method (Figure 1, Table 1). Phase 1 aims to compile current knowledge of guideline adaptation, phase 2 identifies initial methodological principles and generates the initial GDC‐adaptation extension items, phase 3 will iteratively refine the two products and reach consensus on the extension items through GIN adaptation working group meetings and Delphi consensus surveys, respectively, and phase 4 focuses on the public consultation of the methodological principles and user testing of the GDC‐adaptation extension. The Delphi consensus survey will be 2–3 rounds, depending on the consensus and feedback received. We will conduct public consultation through an online survey among GIN organizational members and main stakeholders regarding the methodological principles. We will perform several rounds of user testing with key stakeholders to improve the usability and internal validity of the GDC‐adaptation extension. Finally, we will seek approval from the core working group and GIN board regarding the Methodological principles and the GDC‐adaptation extension. We plan to finish this project in 3 years (Table 2). The final products of this project will consist of approved GIN methodological principles for guideline adaptation and the GIN‐McMaster Guideline (Table 3).

**Table 1:** Summary of steps and outputs.

Phases	Aim	Content	Outputs	Resources and existing work
1: Current knowledge of guideline adaptation				
Scoping review	To compile the current knowledge of guideline adaptation	Definition and rational Methods and key concepts on guideline adaptation Applications, barriers and facilitators of adaptation methods	Initial key steps of the Methodological principles	Existing adaptation approaches Guideline handbooks adaptation methods Updating literature on adaptation approaches Reviewers
2: Initial version of the methodological principles and GDC‐adaptation extension				
2.1 Initial methodological principles for guideline adaptation	To further incorporate experience and suggestions based on adaptation key steps	Discussion within the GIN adaptation working group	Refined methodological principles for guideline adaptation	Adaptation working group meetings Workshop discussions
2.2 Initial GIN‐McMaster checklist extension for guideline adaptation	To GDC‐adaptation extension based on original GIN‐McMaster GDC and adaptation key steps	Idea generation—asking stakeholders to assess checklist themes/items Relevant without changes Relevant but needing changes Missing components regarding guideline adaptation	Themes and topics for the core team to work on	GIN‐conference Guideline interest groups Other workshops
3: Consensus				
Delphi consensus surveys	To iteratively refine the two products (methodological principles and GDC‐adaptation extension) To reach a consensus on the GDC‐adaptation extension items	Delphi consensus survey (2–3 rounds)	Consensus on items and topics Optimized GDC‐adaptation extension	Guideline adaptation experts Methodological community
4. Consultation and finalization				
4.1 Public consultation	To elicit inputs from stakeholders on the methodological principles	Online survey with GIN connects and main stakeholders (authors of adaptation approaches and adapted guidelines)	Engagement of Stakeholders' inputs	GIN connect collaboration Authors contact
4.2 User testing	To user test and improve usability and internal validity of the GDC‐adaptation extension	Semistructured interviews with main stakeholders	Initially validated GDC‐adaptation extension	Stakeholders Guideline adaptation experts
Approval and finalization				
Finalization	To finalize and seek approval of the GIN adaptation methodological principles and GDC‐adaptation extension	Seek approval from the GIN board and core working group	Finalization of the GIN adaptation methodological principles and the GDC‐adaptation extension based on feedback from previous steps	Core working group GIN board

Abbreviations: GDC, guideline development checklist; GIN, guideline international network.

**Table 2:** Timeline of the project.

	2023						2024												2025								
**Months (1–12)**	7	8	9	10	11	12	1	2	3	4	5	6	7	8	9	10	11	12	1	2	3	4	5	6	7	8	9
**Protocol development**																											
Phase 1: Scoping review																											
Search strategy and screening																											
Data extraction																											
Data synthesis								L																			
Framework deductive analysis								L																			
Finalizing initial steps								L	L																		
Manuscript drafting									L	L	L																
Phase 2: Methodological principles and GDC‐adaptation extension																											
Generating initial versions								L	L	L	L	L	L														
Working group discussion							x	x	x	x	x	x	x	L	L	L	D										
Phase 3: Consensus																											
GIN AWG discussions																											
Delphi consensus survey																											
Phase 4: Consultation and user testing																											
Public consultation																											
User testing																						x	x				
Finalization and approval																											

*Note*: Dark orange cells are pending steps, and light orange cells are finished steps.

Abbreviations: AWG, adaptation working group; GDC, guideline development checklist; GIN, guideline international network.

**Table 3:** Preliminary items for the GDC‐adaptation extension topic 1.

Topic 1: Organization, budget, planning and training		
Original items	Item classification	Adaptation items^a^
1. Establish the structure of the guideline development group and determine the roles, tasks and relationships among the various groups to be involved (e.g., oversight committee or body to direct guideline topic selection and group membership, a working group consisting of experts and methodologists to synthesize evidence, a secretariat to provide administrative support, a guideline panel to develop recommendations and stakeholders and consumers for consultation). See also topics 3, 4 and 6	Modified item 1	*An adaptation working group should be established to oversee and facilitate the overall process, including roles for reviewing source guideline(s), providing capacity building for the adaptation process and collaborating with other organizations like source guideline developer(s)*.
2. Perform a thorough assessment of the proposed guideline development project with respect to financial and feasibility issues concerning the guideline development group (e.g., availability of resources to complete the project, expected commitment from guideline panel and staff).	Modified item 2	Perform a thorough assessment of the financial and feasibility issues for the guideline adaptation process, *especially the required adaptation methodological skills of the panel (e.g., expertise on guideline retrieving and assessment skills, guideline development and adaptation methodology, ideally with the support from methodologists of source guidelines), and potential savings through guideline adaptation*.
3. Obtain organizational approval to proceed with the guideline project.	Modified item 3	*Acquire organizational willingness to adapt source guidelines or obtain organizational approval for a guideline adaptation project*. *Willingness of guideline adaptation organization*. *Willingness of source guideline organization (may clarify copyrights and obtain approval or authorization, if needed)*.
4. Prepare a budget for the development of the guideline, outlining the estimated costs for each step (e.g., remuneration of working group members and staff, cost of outsourcing certain tasks to outside organizations or groups, travel expenses and publication and dissemination expenses).	Original item	Original
5. Determine whether guideline panel members will be provided any payment or reimbursement for their time or will work as volunteers.	Original item	Original
6. Obtain or secure funding for the development of the guideline, with attention to conflict‐of‐interest considerations. See also topic 7.	Original item	Original
7. Outline and arrange the administrative support that will be required to facilitate the guideline development process (e.g., a secretariat of the working group to organize and obtain the declaration of interests and to arrange group meetings).	Original item	Original
8. Plan and prepare for training and support that will be required for those involved in the guideline development process (e.g., conflict‐of‐interest–related education or training for guideline panel members, and teaching sessions for patients to be involved in the guideline group). See also topics 4 and 6.	Modified item 8	Plan and prepare for training and capacity building for the guideline adaptation process, *including training on the adaptation process and the standardized methods used by source guidelines, and guideline quality assessment*.
9. Set a timeline for the completion of the guideline and target dates for the completion of milestones in the guideline development process.	Modified item 9	Set a timeline for the completion of the guideline adaptation *concerning potential accelerated steps, e.g., in particular circumstances, the timeline can be shortened, and written commitments from working group members can be obtained at the inception of the adaptation process to increase the likelihood of completion according to the timeline*.
10. Determine what, if any, legal considerations are relevant for the planned guideline (e.g., reimbursement policies for orphan drugs).	Original item	‐
11. To keep the guideline development group on track, prepare a protocol for the entire guideline that can be completed as the project progresses, including an outline of the overall goals and objectives for the guideline, the timeline, task assignments, steps that will require documentation of decisions and the proposed methods for all steps (i.e., those covered in this checklist, such as the methods for forming the guideline group, selection of topics to be covered in the guideline, consensus methods, consultation methods and methods for the evidence search and selection).	Modified item 11	Prepare a protocol for the *guideline adaptation process, emphasizing the decision of which adaptation approach to follow, potential adaptation entry pathways, dissemination and implementation strategies; define process for evaluation of source guideline, evidence and recommendation*.
‐	New	*The adaptation working group should decide on the need for translation through the guideline adaptation process and then make a translation plan according to the local capacity*.

Abbreviation: GDC, guideline development checklist.

a Modifications are marked in italics.

### 3.1 The GDC‐adaptation extension working group

The Extension Working Group will include (1) a core working group, (2) an expert group and (3) a wider audience. We will collect the Conflicts of interest of all participants.

#### 3.1.1 Core working group

The core working group (Y. S., Y. Z., Y. S. A., A. D., E. A. A., P. A. C., H. J. S.) will lead and coordinate the GIN‐McMaster GDC‐adaptation extension development, as well as lead the methodological principles for guideline adaptation. Specifically, the Core working group will be responsible for (1) protocol publication, (2) generating the initial versions of the checklist extension and methodological principles, (3) implementing each step of the process and (4) reporting the findings of each process.

#### 3.1.2 Expert group

The expert group is a multidisciplinary group (8–10 people), including guideline methodological experts or developers with adaptation experience and expertise, as well as GIN organizational members. We will identify participants through (1) the GIN adaptation working group, (2) main authors from guideline adaptation approaches or adapted guidelines, (3) original GIN‐McMaster GDC core team and panel members and (4) GIN organizational memberships. Expert group members will (1) review and provide expert advice for the methodological principles and GDC‐adaptation extension; (2) ensure stakeholder feedback is considered through the project; (3) ensure compliance with the current adaptation process; (4) participate in online or in‐person workshop discussions.

#### 3.1.3 Wider audience

The wider audience, including the Delphi Panel and GIN adaptation working group members, consists of 20–30 participants with profiles similar to those of expert group members. The responsibilities of the wider audience include (1) participating in the online discussion within the GIN adaptation working group and reaching consensus; (2) participating in the Delphi process or online consensus meetings, if necessary, as Delphi panel members; (3) participating in the public consultation or user testing of the GDC‐adaptation extension; (4) reviewing and providing expert advice. We will aim to balance country income, gender and profile representativeness of participants.

### 3.2 Phase 1: Current knowledge of guideline adaptation

We will conduct a scoping review of the current knowledge of guideline adaptation. We will clarify the current definition of guideline adaptation, key steps of the adaptation process, including adaptation approaches and their applications. We will follow standard methods for scoping review, including the JBI Manual for Evidence Synthesis and adhere to PRISMA Extension for Scoping Reviews for reporting.^23^ The process detail was described in a separate protocol (https://osf.io/wq3ja/).

In the scoping review, we will address the below questions:

1. What are the definitions and rationales for guideline adaptation? (Adaptation glossary).
2. What are the key concepts for adaptation (e.g., assessment and selection of source guidelines, assessment of recommendation contents and updating/supplementing of local evidence)? (Adaptation process).
3. What are the applications, barriers and facilitators of adaptation methods used? (Applications).

#### 3.2.1 Literature search and resources

We have identified any individual adaptation approaches in Medline and EMBASE from 2015 to 3 April 2023. Also, we explored grey literature by forward citation search by January 2024. The major data source for the scoping review included previously published systematic reviews on adaptation methods, individual studies of adaptation approaches from both systematic reviews and an updated literature search of the latest systematic review. Furthermore, we hand‐searched guideline development organization websites and conducted an online survey through GIN Connect to inquire about any potential missing adaptation approaches.

#### 3.2.2 Eligibility criteria

We included (1) relevant systematic reviews on any adaptation methods; (2) individual studies on guideline adaptation approaches with detailed steps, including those that used adaptation as a major step for the guideline development or implementation; (3) adapted guidelines that use identified adaptation approaches and 4) methodological research on the use of identified adaptation approaches. We did not exclude reports based on the language of publication. Two calibrated reviewers independently assessed the eligibility criteria based on the title and abstract of retrieved citations. Two reviewers worked independently to confirm eligibility based on the full‐text assessment of selected studies. Discrepancies were solved with a third reviewer.

#### 3.2.3 Data extraction

We extracted data of (1) individual adaptation approaches on objective and definition and key steps to adapt guidelines according to the GIN‐McMaster GDC topics; (2) adapted guidelines regarding adaptation level (e.g., guideline level, recommendation level or evidence level), level of healthcare (primary healthcare, secondary healthcare, tertiary healthcare), tailoring of the approach and time and resources spent and (3) experiences of using the identified approaches, regarding barriers or facilitators, usability or general feedback and measurements used for identifying barriers and facilitators. Another research group is in parallel systematically surveying new adaptation frameworks and adapted guidelines since two systematic surveys,^9^, ^24^ that will also inform the development of the GDC‐extension.

#### 3.2.4 Data analysis

We applied the deductive content analysis for qualitative data. First, two members from the core working group generated an initial list of key steps according to the GIN‐McMaster GDC topics, based on group members' experience, in parallel with the scoping review process. Second, the two members from the core working group reviewed newly identified adaptation approaches and methods, extracted the key steps of the adaptation process and sought additional concepts from the updated literature against the initial list of key steps. The core working group reviewed and discussed the key steps of the adaptation process and agreed on a preliminary version to be included in phase 2.

### 3.3 Phase 2: Initial version of the methodological principles and GDC‐adaptation extension

#### 3.3.1 Initial methodological principles for guideline adaptation

We proposed and described the methodological principles based on the key adaptation steps from phase 1, in parallel, generating the GDC‐adaptation extension items. We then will refine the key steps through core group discussions. First, we will present the key steps as initial methodological principles in the meeting for discussion and feedback collection. Then, the core working group will refine the methodological principles based on the discussion or agreements. We will continuously discuss and refine the methodological principles through core team meetings until an agreement is reached. We will document all the decisions regarding modifying any items or contents with justifications based on discussion within the core working group. Then, we will share a process report with the expert group for feedback and suggestions.

#### 3.3.2 Initial GIN‐McMaster checklist extension for guideline adaptation

In parallel, we are developing the GDC‐adaptation extension by analyzing the original GDC items based on the key steps of the adaptation process and the core working group's discussions. This step aims to generate GDC‐adaptation extension items. With the core working group, we will analyze the key adaptation steps and against them with the original GDC. We will identify and generate the domains and items relevant to the GDC‐adaptation extension. We will assess the relevance of the original checklist domains and items and classify the extension items as (1) modified items with further explanation or inclusion of adaptation contents; (2) new items that are missing from the original GDC but necessary for guideline adaptation; (3) original items that work for both guideline adaptation and de novo guideline development process and 4) irrelevant items for guideline adaptation. We will regularly meet virtually to discuss and iteratively engage the knowledge from step 2.1, including online workshops, group discussions and public consultation, to incorporate stakeholders' inputs and make necessary changes to the original checklist.

We will document all feedback and comments collected through workshops and discussions. Then, the core working group will review the comments received and refine the GDC‐adaptation extension through virtual core working group meetings. All the decisions regarding modifying items will be documented with justifications based on discussion within the core working group. The whole process will be shared with the expert group for any feedback and suggestions.

### 3.4 Phase 3: Stakeholder engagements and consensus

We will conduct two to three rounds of meetings within the GIN Adaptation working group to reach an agreement regarding the methodological principles for guideline adaptation. Each discussion will start with the introduction of the project, the development process of the guideline adaptation methodological principles, results and modifications of each round of discussion and the current version of the methodological principles. If no further concerns or comments are collected, we will proceed with a voting process. At least 80% of participants vote approval will end the consensus process. All the decisions regarding modifying items or the consensus process will be documented with justifications. The whole process will be shared with the expert group for any feedback and suggestions.

Regarding the GDC‐adaptation extension, we will perform several online Delphi consensus surveys to reach an agreement on the GDC‐adaptation extension items.^25^, ^26^ We will invite key stakeholders as Delphi group members, including the main authors from the adaptation approaches and adapted guidelines, main authors from the original GIN‐McMaster GDC with adaptation experience and other stakeholders with guideline adaptation, adoption or de novo development experience in the last 3 years. We will record the Delphi group members' guideline adaptation experience, working organizations and countries' income levels. We will assess participants' consensus using a 7‐point scale, indicating their level of agreement (1 = strongly disagree, 7 = strongly agree),^27^ and a blank box under each item to receive any feedback the participants may have. We will use Survey Monkey for the online Delphi consensus survey (https://www.surveymonkey.com/). After each round of Delphi, we will review and discuss items that receive a median score lower than 6, two members of the core working group will propose potential modifications according to suggestions received. Then, the whole core working group will discuss and agree on the modifications. After two rounds of surveys, items that did not reach consensus (median score lower than six) will be extracted for further discussion. We will iteratively refine the GDC‐adaptation extension until consensus has been reached for each item (median score no less than six). All the decisions regarding modifying items or consensus processes will be documented with justifications. The whole process will be shared with the expert group for any feedback and suggestions.

### 3.5 Phase 4: Consultation and user testing

We will actively seek feedback to finalize both products, from stakeholders who have not been previously involved in the development of the methodological principles and the GDC‐adaptation extension. For methodological principles, this involves public consultation while for GDC‐adaptation extension, we will conduct user testing through semistructured interviews.

#### 3.5.1 Public consultation

We will then conduct public consultation on the refined methodological principles. The public consultation will be processed through an online survey to collect further suggestions on the adaptation methodological principles from GIN organizational members. We will also invite other relevant stakeholders for public consultation, including authors of published adaptation methods and guidelines, or suggestions from expert colleagues. We will record the organizational members' experience regarding guideline development and adaptation, countries' income levels and the specialist components of the guideline methods team.

The survey questionnaire will be designed based on the methodological principles for guideline adaptation. We will collect participants understanding and comments on the current version of the principles, then assess whether the contents are useful, using a 7‐point scale (1 = strongly disagree, 7 = strongly agree). Given the exhaustive process, we will not expect any new components to emerge at this step. If there are any newly emerged criteria, we will ask for supportive evidence (e.g., newly developed adaptation methods or approaches, organizational methodological principles or the self‐administrated adaptation process) and deliberate carefully regarding further inclusion within the core working group. The whole process will be documented and shared with the expert group for any feedback and suggestions.

#### 3.5.2 User testing

This step aims to improve the usability and internal validity of the GDC‐adaptation extension checklist. We will conduct several rounds of semistructured interviews until no major feedback for revision emerges.^28^ We plan to conduct at least three user tests and modify the GDC‐adaptation extension accordingly.

We will recruit user testing participants from any guideline adaptation working groups that have adapted guidelines and/or that have participated in the guideline adaptation in the last 3 years. We will take into account country income level, type of organization and region. Participants' conflict of interest and consent for participation will be obtained before the interview starts.

We will collect feedback through semistructured interviews using an interview guide based on previous user testing studies and our experience.^11^, ^29^, ^30^ We will guide users, through a series of questions using examples to simulate the CPG adaptation process, to explore which parts of the GDC‐adaptation extension cause them concerns, probing to understand the nature of these concerns. The interview will last approximately 40 min. We will audio record the interviews with participants' permission and transcribe the interview script verbatim. We will collect participants' feedback on the domains of the GDC‐adaptation extension, including overall feedback, missing aspects or implementation considerations, potential barriers for guideline adaptation and needs for methodological support. The whole process will be documented and shared with the expert group for any feedback and suggestions.

#### 3.5.3 Data analysis and refinement

For quantitative data, including characteristics of participants and workplaces, Delphi consensus scores and usefulness scores, we will calculate absolute frequencies and proportions. For qualitative data, including comments on each version, participants' understanding of each GDC‐adaptation extension item and methodological principle and feedback received in each user testing, we will conduct a content analysis.^31^ Based on each round, we will further iteratively refine and finalize the GDC‐adaptation extension and methodological principles for final approval.

### 3.6 Approval and finalization

The core working group will compile the final versions of the two products, methodological principles and the GDC‐adaptation extension. We will share the final version of the adaptation methodological principles with the GIN adaptation Working Group members, and the GIN Board, to seek final approval. The core working group will also approve the final version of the GDC‐adaptation extension. We will highlight the changes from the original GIN‐McMaster GDC regarding guideline adaptation, including additional contents, further explanations apart from the original item and new items.

### 3.7 Ethical approval

We obtained an ethical waiver from the institutional review board of the Chinese University of Hong Kong—Shenzhen (exception number: CUHKSZ‐D‐20240032), as no patients, biological samples or clinical data will be collected. However, all data will be stored anonymously, and informed consent will be obtained from all participants. We will also avoid collecting identification information for user testing participants apart from obtaining informed consent. All participation will be voluntary, and all participants will be notified about their right to withdraw at any stage of the interviews. We will document all collected data confidentially, and only the core working group members will have access to the primary data. We will only use interview data for the user testing of the GDC‐adaptation extension items.

## 4 DISCUSSION

The key outputs from the proposed study will include the methodological principles for guideline adaptation and the GIN‐McMaster GDC‐adaptation extension. The key principles will be a minimum set of criteria that guideline adaptation and contextualization processes should follow. The GDC‐adaptation extension will inform and guide adaptation processes of guideline developers with specific steps, links to tools and resources and examples.

### 4.1 Comparison with previous/ongoing research

Although many adaptation frameworks have been developed, to date, few have been used, and diverse experiences of guideline adaptation are still emerging.^32^, ^33^ ADAPTE was one of the first adaptation methods, with a comprehensive toolkit for guideline adaptation, developed based on evidence until 2006 and engaged with invaluable experience from multiple guideline developers and experts.^34^, ^35^ The ADAPTE has been promoted by the GIN community as the standard method for adaptation since 2010.^35^, ^36^, ^37^ Considering guideline development methods of source guidelines became more rigorous,^38^ ADAPTE has been modified on several occasions.^39^, ^40^ In 2018, the GIN adaptation working group recognized the out‐of‐date ADAPTE toolkit, but faced many challenges in updating it, as the guideline development methods have evolved importantly, since the original publication. Another important framework is the GRADE‐ADOLOPMENT,^8^ developed based on the GRADE‐EtD framework and GRADE approach,^41^ which proposes to adapt or develop de novo recommendation following the GRADE EtD framework. This framework is embedded into the GRADEpro software.

Different from previous adaptation frameworks, our current proposal will be based on the existing guideline development checklist—the GIN‐McMaster GDC, engaging inputs from experts in the guideline methodological community (i.e., GIN adaptation working group, interested organizations and authors from adaptation papers), and finally provide a minimum set of methodological principles for guideline adaptation and a comprehensive list for guideline adaptation process (the GIN‐McMaster GDC‐adaptation extension).

### 4.2 Limitations and strengths

We will use multiple techniques (e.g., group consensus and the Delphi method) to gather expert opinions and reach a consensus on the methodological principles and the GDC‐adaptation extension items. However, the consensus methods rely on participants' selection and expertise. To minimize bias and ensure the internal validity, we will invite all GIN adaptation working group members with expertise on the guideline adaptation methods, main authors from adaptation approach papers and experts from the original checklists with sufficient knowledge of the guideline development process. We will not apply the GDC‐adaptation extension or the methodological principles in a real‐life adaptation process, considering the time and project constraints. However, we will test and improve the usability of the GDC‐adaptation extension based on user testing with key stakeholders, which will enhance the validity of the GDC‐adaptation extension. Further study design will need to focus on the external validation of the GDC‐adaptation extension in different contexts.

We will follow a rigorous development process, to ensure the validity of this work, which will be based on scoping reviews of current knowledge, experts' experiences and inputs from user testing. Considering that the adaptation process should follow a similar process as the development of the trustworthy original guideline, our current proposal will be developed based on the existing guideline development checklist—the GIN‐McMaster GDC‐adaptation extension. The main work will provide a comprehensive extension for guideline adaptation, aligning with the validated GIN‐McMaster guideline development checklist. We will also propose key principles for guideline adaptation to standardize and streamline the process.

### 4.3 Implications for practice and research

The methodological principles together, with the GIN‐McMaster GDC‐adaptation extension, will provide clarity in the planning, and execution of adaptation, adoption or development of recommendations. The aim of the checklist is to improve the efficiency of the process and reduce research waste in guideline development while maintaining rigour and transparency. Publication of the principles and GDC‐adaptation extension makes validation by others possible. Ultimately, as in any science, this work may have to be updated.

Different audiences can use the GIN‐McMaster GDC‐adaptation extension. Guideline developers can use the checklist for the entire or core steps of the adaptation process. Educators or researchers, responsible for medical education, can teach the adaptation process to medical doctors or trainees. Policymakers may consider the GDC‐adaptation extension when implementing certain healthcare measures or to fill gaps inside the evidence ecosystem, such as using the GDC‐adaptation extension to adapt a health technology assessment or lists of essential medicines. Clinicians and primary care practitioners may consider the GDC‐adaptation extension when implementing recommendations developed at a higher level (international or national level, etc.). Patients could benefit from the adapted and contextualized recommendations, developed following GDC‐adaptation extension.

GIN has proposed several GIN standards for the guideline development process, such as the GIN standards for CPG in 2012^43^ and the GIN standards for COI in 2016.^42^ Those standards represented the consensus of an international, multidisciplinary group of active guideline developers, provided guidance for its members and other organizations and promoted discussion and eventual agreement on a set of international standards for guideline development.^42^, ^43^ Our work will propose methodological principles for guideline adaptation, engaging inputs from multiple stakeholders, reaching consensus and, finally, may also be approved as a GIN methodological principle for guideline adaptation to benefit the methodological community toward GIN.

The GIN adaptation working group is moving forward to engage more guideline adaptation stakeholders, providing an environment for members to share and exchange ideas. The adaptation methodological principles derived from this proposal will be available to all GIN members as a methodological resource to facilitate future guideline adaptation processes. In the future, we will collect user experience and feedback to periodically update the principles and the GDC‐adaptation extension to benefit the whole guideline community.

## AUTHOR CONTRIBUTIONS

*Conceptualization:* Yang Song, Yuan Zhang, Pablo Alonso‐Coello, and Holger J. Schünemann. *Methodology:* Yang Song, Yuan Zhang, Pablo Alonso‐Coello, Holger J. Schünemann, Yasser Amer, Andrea J. Darzi, and Elie A. Akl. *Writing—original draft:* Yang Song, Yuan Zhang, Pablo Alonso‐Coello, and Holger J. Schünemann. *Writing—review & editing:* Yang Song, Yuan Zhang, Pablo Alonso‐Coello, Holger J. Schünemann, Yasser Amer, Andrea J. Darzi, and Elie A. Akl. *Investigation:* Yang Song, Yuan Zhang, Pablo Alonso‐Coello, Holger J. Schünemann, Yasser Amer, Andrea J. Darzi, and Elie A. Akl. *Visualization:* Yang Song, Yuan Zhang, and Holger J. Schünemann.

## CONFLICT OF INTEREST STATEMENT

Dr. Yasser Amer and Prof. Holger J. Schünemann are part of the Editorial Board of Clinical and Public Health Guidelines but did not participate in the editorial process of this article and had no influence on the editorial decisions related to it. All core team members are from the GIN board or the GIN adaptation working group to ensure representativeness; Drs. Yuan Zhang and Pablo Alonso‐Coello, Profs. Elie A. Akl and Holger J. Schünemann are the main authors of the GIN‐McMaster guideline development checklist. The remaining authors declare no conflict of interest.

## ETHICS STATEMENT

Our study did not involve patients and publics.

## Data Availability

All data of this protocol are available upon reasonable request. The contact person is Yang Song (songyang@cuhk.edu.cn).
